# Impact of cigarette smoking on early complications after liver transplantation: A single-center experience and a meta-analysis

**DOI:** 10.1371/journal.pone.0178570

**Published:** 2017-05-30

**Authors:** Qingshan Li, Yue Wang, Tao Ma, Xuemin Liu, Bo Wang, Zheng Wu, Yi Lv, Rongqian Wu

**Affiliations:** Shaanxi Provincial Center for Regenerative Medicine and Surgical Engineering, Institute of Advanced Surgical Technology and Engineering, Department of Hepatobiliary Surgery, First Affiliated Hospital of Xi’an Jiaotong University, Xi’an, Shaanxi Province, China; University of Toledo, UNITED STATES

## Abstract

**Background:**

While studies have shown that cigarette smoking has negative implications on the long-term outcome following liver transplantation, its role in early complications is inconclusive.

**Methods:**

The clinical data of 162 consecutive adult patients who underwent elective liver transplantation from January, 2012 to March, 2016 were analyzed. Patients were defined as active smokers, ex-smokers, or non-smokers on the basis of documentation at the time of liver transplantation. The overall complications following liver transplantation were expressed as the comprehensive complication index (CCI). The specific complications such as the incidence of hepatic artery thrombosis, biliary complications, acute kidney injury were also assessed. A meta-analysis was carried out based on results from the present study and 11 published studies.

**Results:**

We found that cigarette smoking was not associated with higher CCI scores and smokers did not have a higher risk for developing hepatic artery thrombosis, biliary complications, acute kidney injury after liver transplantation. Meta-analysis confirmed the null association between cigarette smoking and an increased incidence of hepatic artery thrombosis or biliary complications in liver transplant recipients. However, the pooled results showed a significantly higher risk of cardiovascular diseases and de-novo malignancies in smokers following liver transplantation.

**Conclusion:**

There is not enough evidence supporting an association between cigarette smoking and early mortality and morbidity after liver transplantation. However, smokers should still be encouraged to quit before and after liver transplantation due to the long-term health benefits of smoking cessation.

## Introduction

Cigarette smoking is the most common form of substance use worldwide. The deleterious sequelae of tobacco use are well recognized and the inherent health benefits of smoking cessation cannot be overstated. According to the World Health Organization, cigarette smoking was responsible for the death of 100 million people worldwide in the 20^th^ century. And it remains a major public health problem in the 21^st^ century[1]. Tobacco use is quite prevalent among liver transplant patients, with a reported range of 14.7% to 75%[2–5]. However, the potential relevance of cigarette smoking to morbidity and mortality following liver transplantation is understudied. Although cigarette smoking was found to be associated with serious long-term negative consequences including de novo malignancies and deceased patient and graft survival following liver transplantation[6–9], its role in early complications was inconclusive.

Clinically, whether to use abstinence from smoking as a transplant selection criterion for liver transplantation remains an ethical challenge[10–12]. The key issue is to understand the true impact of smoking prior to transplantation on outcomes, especially early mortality and morbidity, after liver transplantation. In a 2010 review, Bright RP evaluated the medical evidence on whether the denial of transplantation to smokers is ethical[10]. He found that most of the studies demonstrated increased morbidity and mortality after liver transplantation among smokers. Similarly, a more recent review by Corbett et al showed that active smoking was associated with increased risk of hepatic artery thrombosis, biliary complications, and malignancy in liver transplant patients[13]. However, the reviews did not make a clear distinction between pre- and post-transplant smoking. Moreover, they both admitted that not all studies supported their conclusion. More specifically, a retrospective study of 2260 patients with chronic liver disease who were evaluated for liver transplantation at the University of Michigan has shown that smoking was not associated with increased mortality risk at any time point in those evaluated or receiving transplants[12]. Unfortunately, neither of the reviews used meta-analytic techniques.

Duerinckx et al. conducted a meta-analysis to investigate the correlates and outcomes associated with post-transplant smoking after solid organ transplantation[14]. They found that post-transplant smoking was associated with higher odds of newly developed cardiovascular disease, de novo malignancies, and a shorter survival time after liver transplantation. However, the impact of pre-transplant smoking on early post-transplant complications remained unclear. The purpose of this study was to thoroughly investigate the effects of smoking prior to transplantation on early mortality and morbidity after liver transplantation. We first analyzed the clinical data of 162 consecutive adult patients who underwent elective liver transplantation in our center and then conducted a meta-analysis to summarize results from the literature and the current study.

## Methods

### Patients and data acquisition

We retrospectively reviewed the records of 162 consecutive adult patients (18 years of age or older) who received donation after cardiac death (DCD) and underwent elective liver transplantation at the First Affiliated Hospital, Xi’an Jiaotong University School of Medicine from January, 2012 to March, 2016. Clinical data were gathered on all transplant recipients from our institutional electronic medical record, including demographics features, smoking status, perioperative laboratory values and postoperative complications. Five patients did not have smoking status available and were excluded from further analysis. The remaining 157 patients composed our study population. This study was approved by the institutional review board of the First Affiliated Hospital, Xi’an Jiaotong University School of Medicine. For this type of study formal consent is not required.

### Definition of smoking behaviors

Tobacco use status was determined from the review of all clinical encounters the subject had at our center at the time of liver transplantation, and was self-reported by all recipients. Smoking cessation was recorded based on the self-report of the transplant recipient. “Active smoker” included anyone actively smoking at the time of liver transplantation or who had quit for less than 3 months before liver transplantation. “Ex-smoker” included anyone who had previously smoked routinely but had quit for at least 3 month before liver transplantation. “Non-smoker” included patients without any history of regular smoking[15]. Total tobacco exposure for all active and ex-smokers was assessed using “pack-years” which is calculated by multiplying the average number of packs of cigarettes smoked per day by the number of years smoked. For analysis, 2 pack-year groups were constructed, < 20 years was defined as light smokers, while ≥ 20 years was defined as severe smokers. No laboratory surveillance was conducted to confirm self-reported smoking status. There was no posttransplant prospective monitoring of smoking status because this was a retrospective study.

### Definitions of outcomes

Postoperative complications were defined as a diagnosis of complications and mortality within 90 days after transplantation. The primary outcome was the comprehensive complication index (CCI). The CCI was recently developed to document postoperative complications. It measures surgical morbidity by adding up all complications attributable to a surgical procedure and weighting them according to their severity. Thus the CCI reflects the summative severity of all major and minor postoperative complications in a single patient. By avoiding underreporting minor complications, the CCI is a robust system to evaluate postoperative morbidity. Due to its consistency and completeness, the CCI has become one of the standard ways to report postoperative complications in clinical trials. The CCI score ranges from 0 (no complications) to 100 (death). Patients with a CCI score higher than 30 are considered to have a severe postoperative condition. [16]. The secondary outcomes included the incidence of hepatic artery thrombosis, biliary complications, acute kidney injury, as well as lengths of ICU and hospital stay and in-hospital mortality after liver transplantation.

### Statistical analysis

Continuous data was tested for normality by the Kolmogorov-Smirnov test. Normal distribution variables are reported as means ± standard deviations (SD) and compared by the student’s t-test (for 2 groups) or one-way analysis of variance (ANOVA) using the Fisher LSD post hoc method (for 3 groups). Abnormal distribution variables are reported as medians (interquartile range, IQR) and compared by the Mann-Whitney rank-sum test (for 2 groups) or Kruskal-Wallis one-way ANOVA on Ranks using the Nemenyi method (for 3 groups). Categorical variables are reported as numbers and percentages and compared by the Chi-squared analysis or Fisher’s exact test. P<0.05 was considered to be statistically significant. All statistics analyses were done using the IBM SPSS (version 20.0).

### Meta-analysis

The meta-analysis was performed in accordance with Preferred Reporting Items for Systematic Reviews and Meta-Analyses (PRISMA) guidelines[17]. We followed the Meta-analysis of Observational Studies in Epidemiology (MOOSE) consensus in this meta-analysis[18].

1) Search Strategy: Pubmed, Sciencedirect, and Web of Science were searched systematically to identify all available studies that examined the associations between cigarette smoking and post-operative complications after liver transplantation using the following key words: smoking (“tobacco use” or “cigarette smoking”), liver transplantation and outcome (“complication” or “morbidity”). The search was completed on May 5, 2016. No language restrictions were imposed. All references cited in those relevant studies were also reviewed.

2) Study Selection: Studies were considered suitable for inclusion if they met the following criteria: a) human studies with participants older than 18 years old undergoing liver transplantation; b) they reported the association of smoking with postoperative morbidity (Cardiovascular disease, Hepatic artery thrombosis, Malignancy, Biliary complication); c) relative risk (RR) with its 95% confidence interval (95% CI) could be calculated for any of the outcomes; d) full text available. If we could not obtain this information, the study was excluded from the analysis.

3) Data Extraction and Quality Assessment: Relevant study information (the first author name, year of publication, study design, country, number of subjects, the mean age of subjects, sex distribution, the RRs and the corresponding CI.) was extracted from the publications. Study quality was independently assessed with Newcastle-Ottawa quality assessment scale (NOS) according to the Cochrane Non-Randomized Studies Methods Working Group. This instrument uses a “star system” to evaluate data quality. The system criteria included three broad perspectives: the selection (four stars), comparability (two stars) and outcome (three stars); the quality scores of studies range from zero (lowest) to nine (highest). A score of five or greater was considered high quality, whereas scores less than four were considered low quality[19].

4) Statistical Analysis: Combined RR with its corresponding 95% CI was used to measure the impact of cigarette smoking on post-operative complications after liver transplantation. The heterogeneity across studies was evaluated by the Q test and I2 statistics (I2 > 50% indicated evidence of heterogeneity). The meta-analysis, applying the random-effect model, was carried out using STATA (version 12.0, StataCorp, College Station, TX, USA). We further performed sensitivity analysis by sequential omission of individual studies or by omitting studies without high quality. In addition, funnel plots were applied in order to assess the potential publication bias. The analysis was conducted independently and in a double-blind manner by two investigators (Qingshan Li and Tao Ma). A P value<0.05 was considered statistically significant, except where otherwise specified.

## Results

### Smoking prevalence

There were 157 patients in this retrospective study. Of the 44 patients (28.0%) with a positive smoking history, 38 were active smokers (24.2%) and 6 were ex-smokers at the time of liver transplantation. The remaining 113 patients (72.0%) reported life-long non-smoking. Therefore, the prevalence of active smoking in our cohort is similar to that observed in the general population.

### Baseline and intra-operative characteristics

Most of the baseline and intra-operative variables were similar among groups. Baseline demographic data are documented in Table 1. However, smokers were more likely to be men than women (p = 0.005). Active smokers also had considerably higher plasma levels of creatinine (p = 0.004) and blood urea nitrogen (p = 0.04) than non-smokers. Intra-operative factors are shown in Table 2. The operation time was significantly longer in patients with a positive smoking history (p = 0.037) as compared with patients who never smoked. Active smokers also experienced a longer Anhepatic phase (p = 0.009) than non-smokers.

**Table 1:** Characteristics of demographic and clinical features of the patients, according to smoking status and pack-years exposure.

Variables	Smoking status				pack-years exposure			
	Active smoker (N = 38;24.2%)	Ex-smoker (N = 6;3.8%)	Non-smoker (N = 113;72.0%)	P value	Severe smoker (N = 15;9.5%)	Light smoker (N = 29;18.5%)	Non-smoker (N = 113;72.0%)	P value
**Demographic features**								
Age (years)	47.37±10.92	53.17±6.05	44.67±10.48	0.081	54.6±6.91	44.83±10.62	44.67±10.48	0.002
Gender(male, female)	37/1	6/0	85/28	0.005	15/0	28/1	85/28	0.005
**Clinical features**								
** Preoperative laboratory values**								
Hematocrit (%)	30.4 (24.8, 33.3)	36.7 (25.6, 44.9)	29.9 (26.5, 36.4)	0.529	28.6 (23.25, 36.57)	31.2 (26.7, 34.3)	29.9 (26.5, 36.4)	0.795
Creatinine (μmol/L)	63.0 (49.9, 73.7)	72.1 (69.8, 82.3)	56.0 (46.3, 68.5)	0.004	67.0 (63.0, 97.3)	62.3 (48.9, 73.0)	56.0 (46.3, 68.5)	0.002
Total bilirubin (μmol/L)	31.8 (21.5, 87.8)	31.1 (11.8, 100.9)	49.3 (20.7, 113.5)	0.317	28.43 (19.30, 219.60)	33.53 (19.63, 84.45)	49.35 (20.69, 113.46)	0.435
Red cell (×10^12^/L)	3.19±0.86	3.95±1.12	3.29±0.78	0.102	3.23±1.09	3.33±0.84	3.29±0.78	0.938
Hemoglobin (g/L)	102.5 (83.7, 118.3)	133.5 (96.7, 154.3)	100.0 (89.0, 121.0)	0.104	103 (83, 130)	103 (90, 115)	100 (89, 121)	0.967
Platelet (×10^9^/L)	53.0 (39.5, 89.3)	58.5 (44.5, 117.7)	58.0 (33.0, 99.0)	0.799	63 (37, 105)	52 (41, 67)	58 (33, 99)	0.727
Leukocyte (×10^9^/L)	4.80 (2.50, 6.40)	4.81 (3.67, 5.30)	3.62 (2.43, 5.67)	0.329	5.11 (3.85, 6.95)	4.61 (2.49, 5.67)	3.62 (2.43, 5.67)	0.118
Lymphocyte (×10^9^/L)	0.70 (0.43, 1.18)	1.13 (0.52, 1.43)	0.63 (0.45,1.00)	0.381	1.04 (0.53, 2.24)	0.64 (0.43, 0.99)	0.63 (0.45, 1.00)	0.14
Monocyte (×10^9^/L)	0.37 (0.23, 0.61)	0.39 (0.15, 0.68)	0.33 (0.17, 0.53)	0.417	0.54 (0.27, 0.91)	0.33 (0.21, 0.47)	0.33 (0.17, 0.53)	0.108
Neutrophils granulocyte (×10^9^/L)	2.70 (1.70, 4.81)	2.80 (2.23, 4.41)	2.51 (1.68, 4.65)	0.697	2.24 (2.18, 4.62)	2.85 (1.45, 4.87)	2.51 (1.68, 4.65)	0.741
AFP (μg/L)	3.94 (2.85, 37.39)	12.95 (7.66, 93.95)	3.84 (2.30, 10.02)	0.066	9.26 (3.07, 89.55)	4.26 (2.51, 21.67)	3.84 (2.30, 10.02)	0.13
ALT (U/L)	27.72 (19.50, 46.13)	64.5 (26.99, 88.72)	31.85 (23.35, 56.75)	0.21	38.3 (20.0, 52.0)	27.4 (20.3, 46.6)	31.9 (23.3, 56.70)	0.536
AST (U/L)	38.86 (29.50, 61.56)	44.69 (39.89, 67.25)	46.00 (30.42, 75.61)	0.368	41.0 (34.0, 87.0)	39.0 (30.7, 53.2)	46.0 (30.4, 75.6)	0.469
Albumin (g/L)	33.60 (28.97, 36.83)	35.05 (25.42, 42.55)	25.61 (31.23, 41.93)	0.165	33.40 (29.42, 36.21)	33.74 (28.95, 38.92)	35.61 (31.23, 41.93)	0.167
BUN (mmol/L)	5.31 (4.15, 7.81)	5.04 (3.83, 6.31)	4.53 (3.32, 6.10)	0.04	5.07 (3.98, 9.40)	5.51 (4.18, 7.13)	4.53 (3.32, 6.10)	0.053
Lactate (mmol/L)	1.60 (1.03, 2.20)	1.15 (1.10, 1.65)	1.40 (1.00, 2.20)	0.857	1.65 (1.05, 1.930)	1.45 (1.07, 2.00)	1.40 (1.00, 2.20)	0.982
Glucose (mmol/L)	5.38 (4.63, 7.09)	8.60 (5.92, 14.31)	5.55 (4.65, 7.40)	0.107	5.36 (4.50, 8.60)	5.92 (4.92, 6.97)	5.55 (4.65, 7.40)	0.94
PT (s)	18.50 (16.65, 21.20)	15.60 (14.85, 17.72)	18.20 (15.50, 22.20)	0.221	19.3 (17.7, 22.2)	17.1 (16.0, 20.7)	18.2 (15.5, 22.2)	0.429
INR	1.58 (1.37, 1.81)	1.31 (1.20, 1.53)	1.51 (1.22, 1.91)	0.362	1.66 (1.50, 1.85)	1.40 (1.30, 1.72)	1.51 (1.22, 1.91)	0.405
APTT (s)	44.6 (40.7, 49.4)	38.3 (34.1, 47.4)	43.4 (39.2, 49.7)	0.242	45.9 (40.9, 49.5)	43.5 (38.3, 49.1)	43.4 (39.2, 49.7)	0.892
** Hepatic features**								
MELD	12.0 (6.0, 16.5)	8.5 (3.5, 14.7)	10.0 (6.0, 15.5)	0.698	13 (9, 22)	9 (6, 13)	10 (6, 15)	0.197
** Etiology**				0.214				0.354
Viral hepatitis	20 (52.6)	3 (50)	53 (47.3)		7 (46.7)	16 (55.2)	53 (47.3)	
Alcoholic cirrhosis	2 (5.3)	0 (0)	3 (2.7)		0 (0)	2 (6.9)	3 (2.7)	
Hepatocellular Carcinoma	14 (36.8)	3 (50)	34 (30.4)		7 (46.7)	10 (34.5)	34 (30.4)	
Primary biliary cirrhosis & Autoimmune liver disease	0 (0)	0 (0)	12 (10.7)		0 (0)	0 (0)	12 (10.7)	
Other	2 (5.3)	0 (0)	10 (8.9)		1 (6.7)	1 (3.4)	10 (8.6)	
** Coexisting conditions**								
Drinking(%)	18 (47.4)	5 (83.3)	7 (6.2)	<0.001	9 (60)	14 (48.3)	7 (6.2)	<0.001
Hypertension(%)	3 (7.9)	0 (0)	5 (4.4)	0.594	2 (13.3)	1 (3.4)	5 (4.4)	0.305
Diabetes(%)	2 (5.3)	1 (16.7)	12 (10.6)	0.52	2 (13.3)	1 (3.4)	12 (10.6)	0.439

Abbreviations: AFP, alpha fetoprotein; ALT, Alanine aminotransferase; AST, Aspartate transaminase; BUN, blood urea nitrogen; PT, prothrombin time; APTT, activated partial thromboplastin time; INR, international normalized ratio; MELD, model for end-stage liver disease.

Normal distribution variables are reported as means ± standard deviations (SD) and compared by the student’s t-test. Abnormal distribution variables are reported as medians (interquartile range, IQR) and compared by the Mann-Whitney rank-sum test. Categorical variables are reported as numbers and percentages and compared by the Chi-squared analysis or Fisher’s exact test.

**Table 2:** Characteristics of intraoperative factors of the patients, according to smoking status and pack-years exposure.

Variables		Smoking status			pack-years exposure			
	Active Smoker (N = 38)	Ex-smoker (N = 6)	Non-smoker (N = 113)	P value	Severe smoker (N = 15)	Light smoker (N = 29)	Non-smoker (N = 113)	P value
Operation time (min)	420 (375, 465)	435 (356, 491)	375 (330, 440)	0.037	435 (390, 485)	405 (363, 450)	375 (330, 440)	0.013
Anhepatic phase (min)	59.32±12.38	51.4±7.16	51.7±11.98	0.009	57.4±9.1	58.6±13.3	51.7±12.0	0.022
Intraoperative blood loss (ml)	2000 (1000, 4000)	3500 (1187, 5000)	2000 (1000, 4000)	0.503	1700 (1000, 4000)	2500 (1200, 5000)	2000 (1000,4000)	0.479
Total input quantity (ml)	7245 (5750, 9712)	7730 (5575, 8975)	6700 (5095, 8365)	0.335	7710 (6360, 10500)	7230 (5590, 8960)	6700 (5095, 8365)	0.224
Warm ischemia time (min)	8 (8, 10)	8 (8, 10)	8 (8, 10)	0.355	8 (8, 11)	8 (8, 10)	8 (8, 10)	0.974
Cold ischemia time (h)	5 (4, 4.5)	6 (4.5, 6)	5 (4, 6)	0.296	4 (4, 5)	5 (4, 6)	5 (4, 6)	0.33

Normal distribution variables are reported as means ± standard deviations (SD) and compared by the student’s t-test. Abnormal distribution variables are reported as medians (interquartile range, IQR) and compared by the Mann-Whitney rank-sum test. Categorical variables are reported as numbers and percentages and compared by the Chi-squared analysis or Fisher’s exact test.

### Influence of smoking on outcomes

Clinical outcomes stratified by smoking status are shown in Table 3. A total of 110 patients developed different postoperative complications according to the Clavien-Dindo system. The median CCI, a novel continuous scale that has been proposed recently to measure surgical morbidity, was 27.2, 20.9, 20.9 in the ex-smoker group, active smoker group and non-smoker group, respectively. The difference was no statistically significant among groups. In terms of the specific complications, smoking did not increase the incidence of hepatic artery thrombosis, biliary complications, acute kidney injury, and ventilation after liver transplantation. Similarly, no significant difference was found among groups in hospital stay, ICU stay, prolonged ICU stay, and postoperative hospital stay as well. Table 4 shows the postoperative outcomes stratified by pack-years of smoking. A total of 110 patients (70.1%) developed postoperative complications, 12 occurred in the severe smoker group, 19 in the light smoker group, and 79 in non-smoker group. This difference was not statistically significant among groups. While in the severe smoker group, the incidence of ventilation was significantly higher than that in the non-smoker group (p = 0.04). No significant difference was found in hospital stay, ICU stay, and postoperative hospital stay based on different pack-years exposure history.

**Table 3:** Postoperative complications, according to smoking status.

Variable	Active smoker (N = 38)	Ex-smoker (N = 6)	Non-smoker (N = 113)	P value		
				Ex-smoker vs. Non-smoker	Active Smoker vs. Non-smoker	Ex-smoker vs. Active Smoker
CCI	20.92(0, 29.58)	27.22(15.69, 34.72)	20.92(0, 31.56)	0.432	0.818	0.438
In-hospital mortality n(%)	2 (5.3)	0 (0)	4 (3.5)	>0.999	>0.999	>0.999
Length of ICU stay (days)	6 (5, 8)	9 (6, 10)	6 (5, 9)	0.169	0.398	0.094
Length of hospital stay (days)	23 (16, 29)	27 (20, 34)	20 (15, 27)	0.16	0.445	0.321
Prolonged ICU stay n(%)	11 (28.9)	4 (66.7)	40 (35.4)	0.266	0.467	0.178
Postoperative hospital stay (days)	19 (14, 25)	21 (14, 34)	19 (14, 24)	0.346	0.971	0.441
Biliary complication n(%)	5 (13.2)	0 (0)	14 (12.4)	0.464	>0.999	0.645
Hepatic artery thrombosis n(%)	0 (0)	0 (0)	4 (3.7)	>0.999	0.554	/

Abbreviations: CCI, comprehensive complication index; ICU, ICU, intensive care unit.

Prolonged ICU stay: postoperative stay in ICU for more than 7 days.

Normal distribution variables are reported as means ± standard deviations (SD) and compared by the student’s t-test. Abnormal distribution variables are reported as medians (interquartile range, IQR) and compared by the Mann-Whitney rank-sum test. Categorical variables are reported as numbers and percentages and compared by the Chi-squared analysis or Fisher’s exact test.

**Table 4:** Postoperative complications, according to pack-years exposure.

Variable	Severe smoker (N = 15)	Light smoker (N = 29)	Non-smoker (N = 113)	P value		
				Severe smoker vs. Non-smoker	Light smoker vs. Non-smoker	Severe smoker vs. Light smoker
**CCI**	20.92 (20.92, 29.58)	20.92 (0, 33.54)	20.92 (0, 31.56)	0.532	0.864	0.769
In-hospital mortality (no/yes)	14/1	28/1	109/4	0.469	>0.999	>0.999
Length of ICU stay (days)	7 (5, 11)	6 (4, 8)	6 (5, 9)	0.349	0.278	0.134
Length of hospital stay (days)	26 (15, 30)	22 (16, 31)	20 (15, 27)	0.384	0.373	0.872
Prolonged ICU stay (no/yes)	8/7	21/8	73/40	0.395	0.428	0.206
Postoperative hospital stay (days)	23 (15, 26)	19 (13, 24)	19 (14, 24)	0.476	0.891	0.457
Biliary complication (no/yes)	15/0	24/5	99/14	0.315	0.705	0.227
Hepatic artery thrombosis (no/yes)	15/0	29/0	109/4	>0.999	0.582	/

Abbreviations: CCI, comprehensive complication index; ICU, intensive care unit.

Prolonged ICU stay: postoperative stay in ICU for more than 7 days.

Normal distribution variables are reported as means ± standard deviations (SD) and compared by the student’s t-test. Abnormal distribution variables are reported as medians (interquartile range, IQR) and compared by the Mann-Whitney rank-sum test. Categorical variables are reported as numbers and percentages and compared by the Chi-squared analysis or Fisher’s exact test.

### Meta-analysis

**Fig 1:**
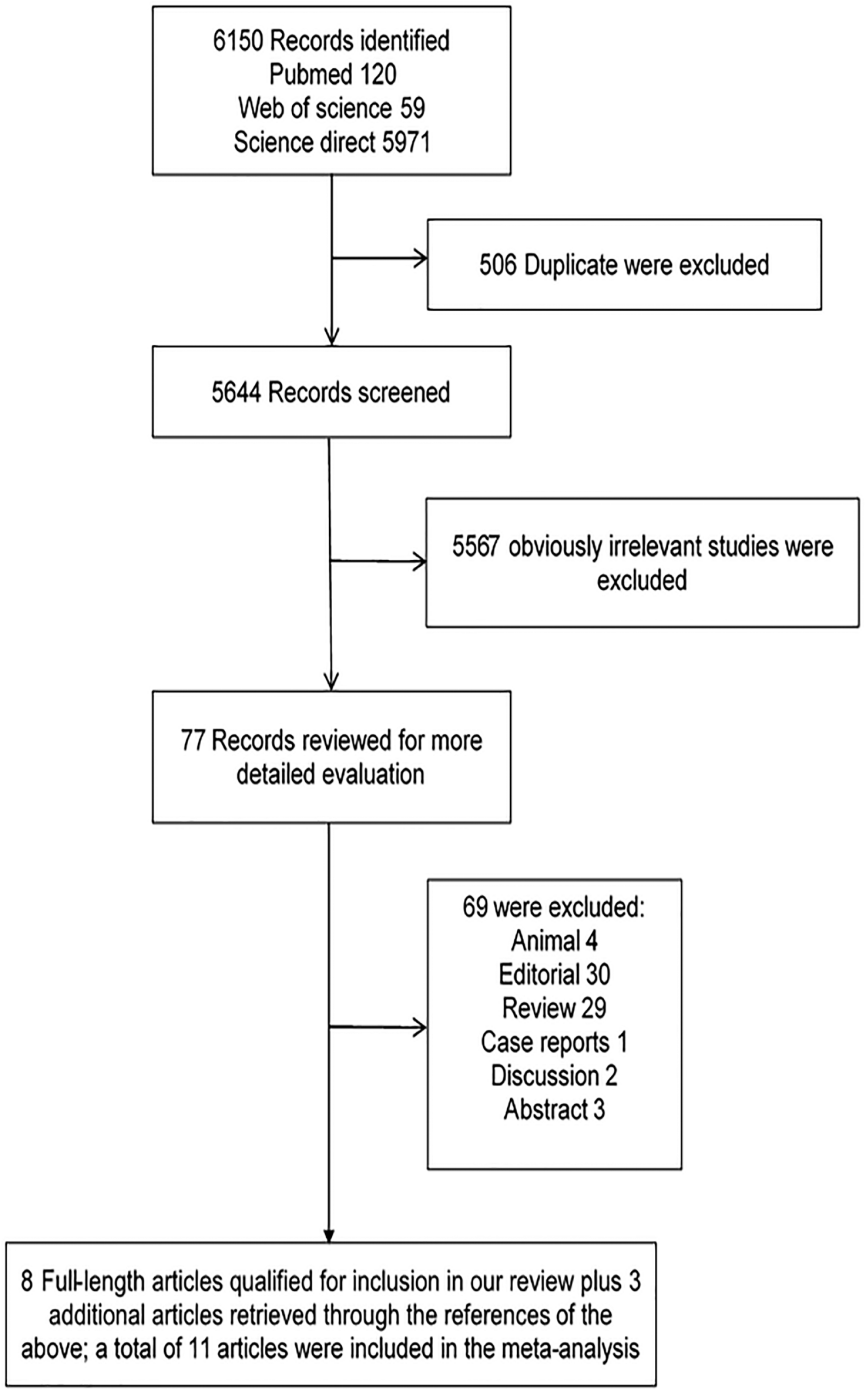
Flow diagram of study selection.

**Fig 2:**
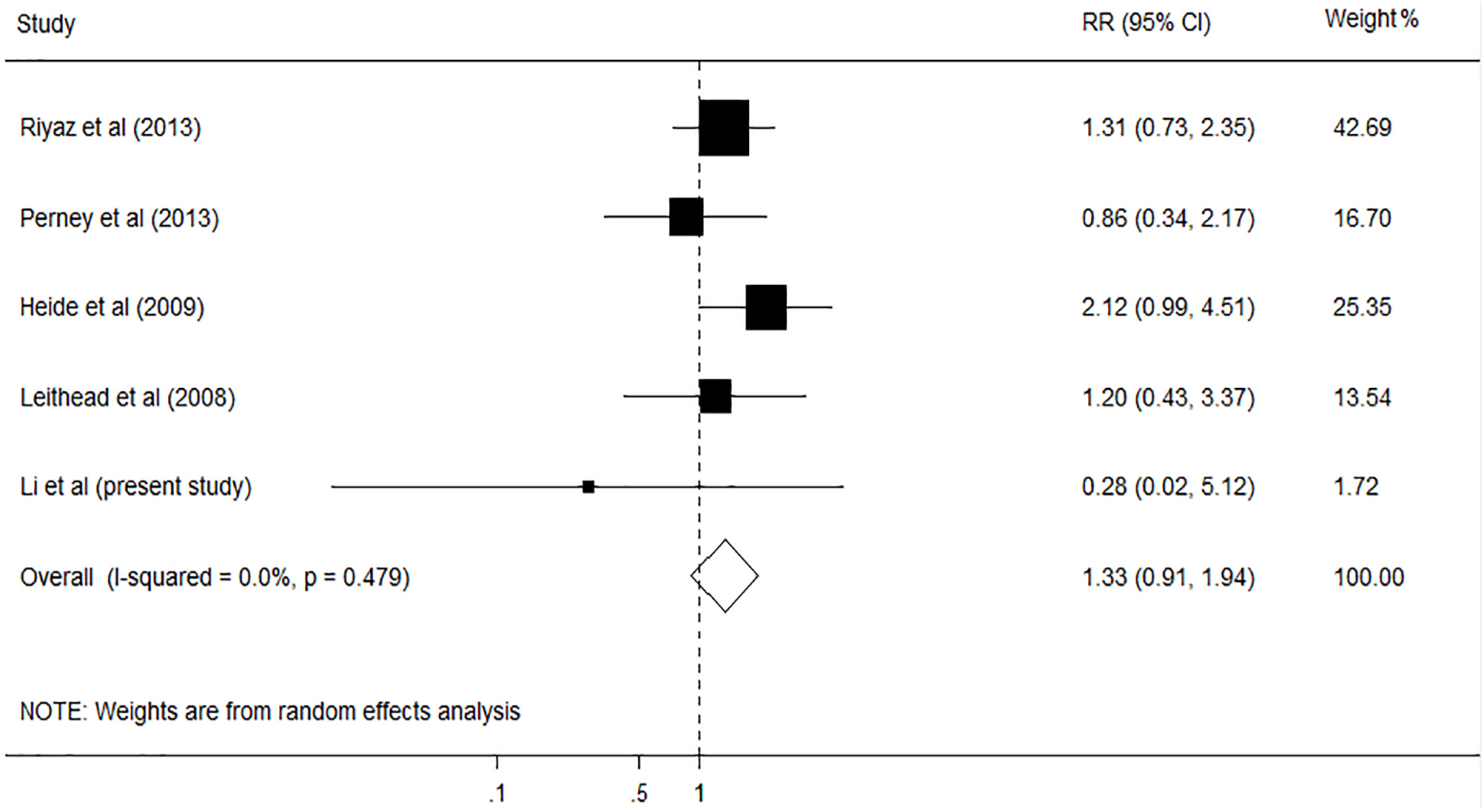
Forest plot on the associations between cigarette smoking and hepatic artery thrombosis after liver transplantation. The boxes and lines indicate the relative ratios (RRs) and their confidence intervals (CIs) on a log scale for each study. The pooled RR is represented by a diamond. The size of the black squares indicates the relative weight of each estimate.

**Fig 3:**
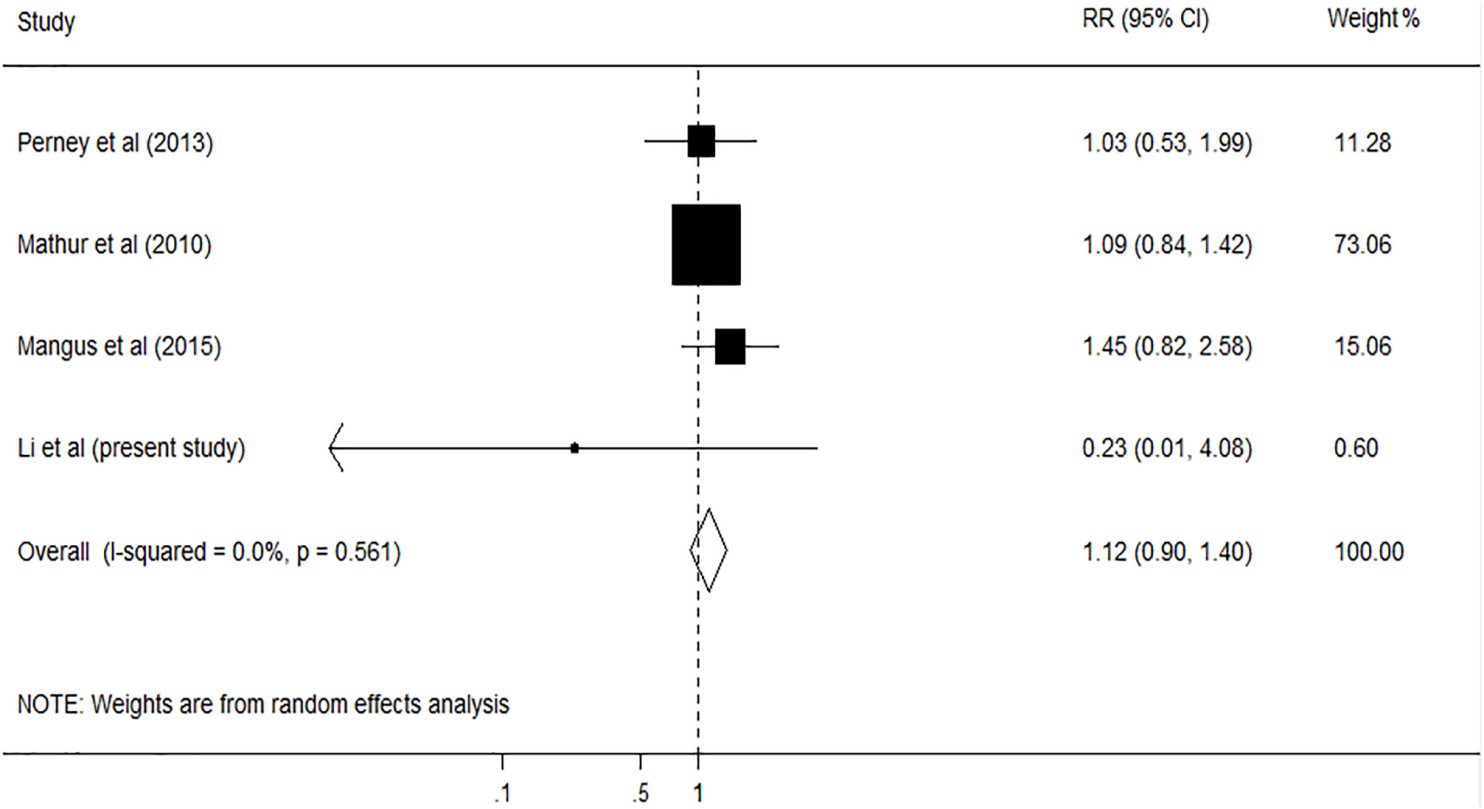
Forest plot on the associations between cigarette smoking and biliary complication after liver transplantation. The boxes and lines indicate the relative ratios (RRs) and their confidence intervals (CIs) on a log scale for each study. The pooled RR is represented by a diamond. The size of the black squares indicates the relative weight of each estimate.

**Fig 4:**
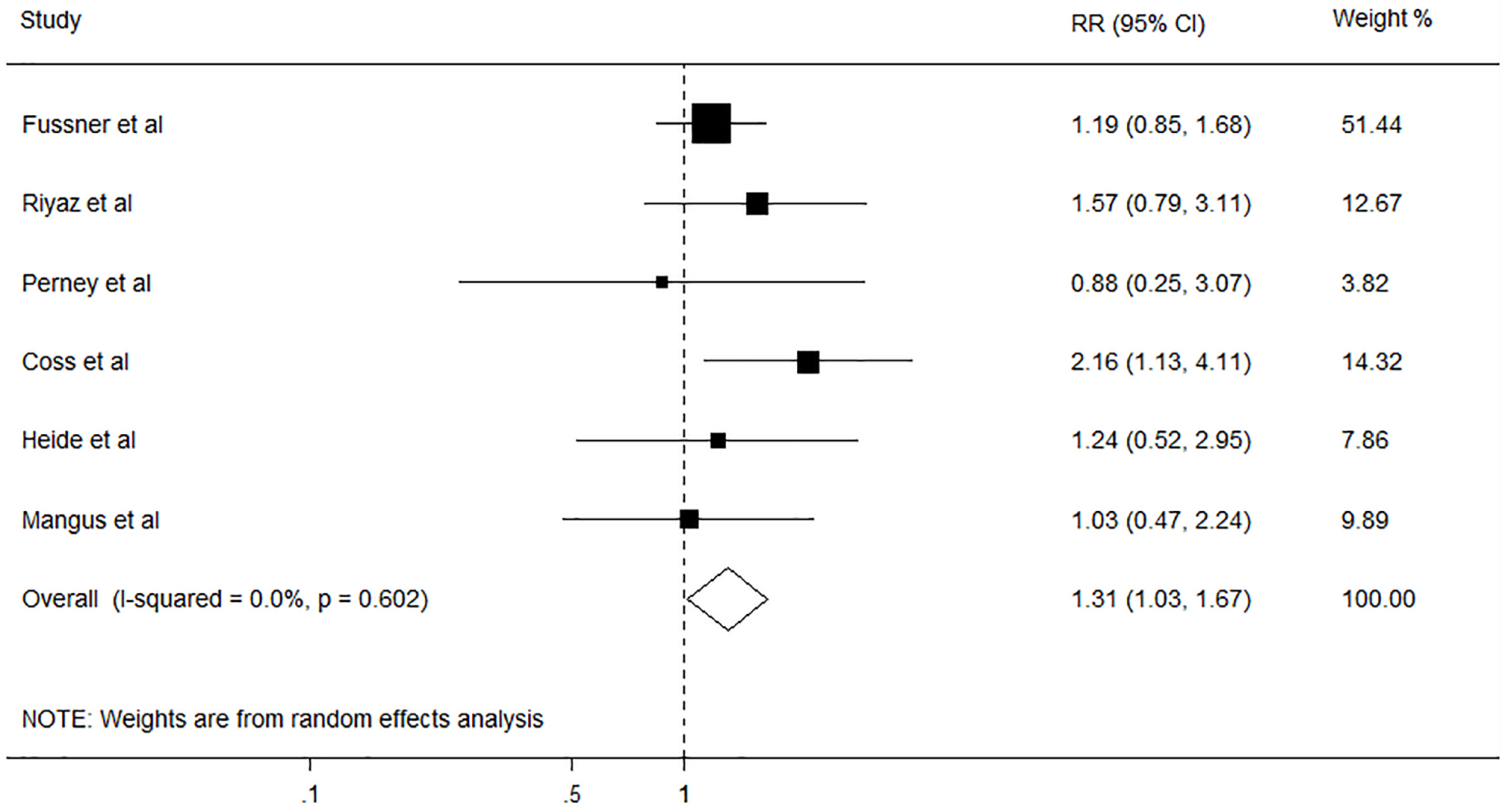
Forest plot on the associations between cigarette smoking and cardiovascular diseases after liver transplantation. The boxes and lines indicate the relative ratios (RRs) and their confidence intervals (CIs) on a log scale for each study. The pooled RR is represented by a diamond. The size of the black squares indicates the relative weight of each estimate.

**Fig 5:**
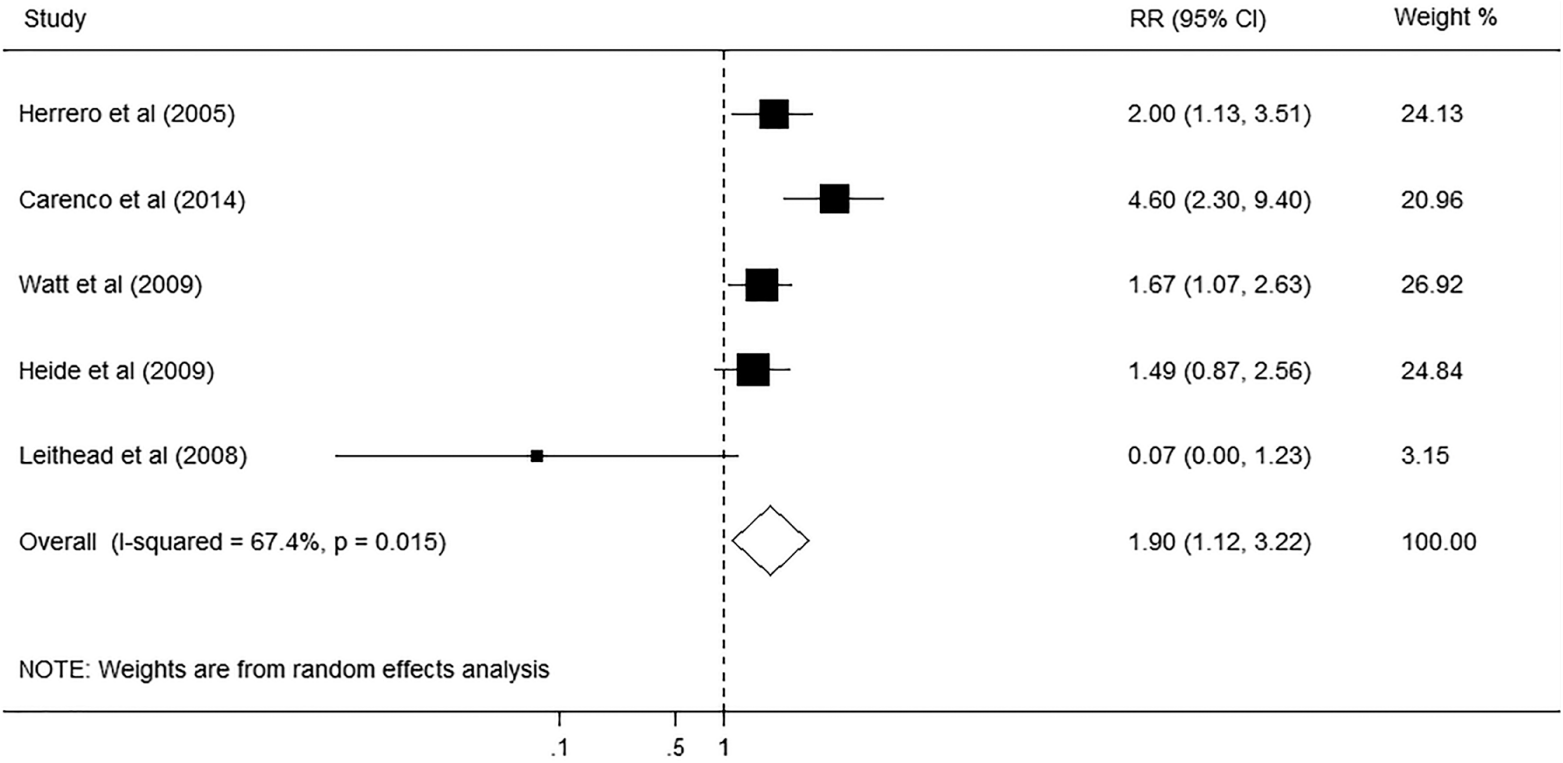
Forest plot on the associations between cigarette smoking and de-novo malignancies after liver transplantation. The boxes and lines indicate the relative ratios (RRs) and their confidence intervals (CIs) on a log scale for each study. The pooled RR is represented by a diamond. The size of the black squares indicates the relative weight of each estimate.

A total of 6150 articles were initially identified from the databases based on our search criteria. After de-duplication, 5644 articles were left for screening according to the titles and abstracts, with 78 records determined as potentially eligible. The full texts and data integrity of these studies were reviewed, and 8 articles and 3 additional articles retrieved through the references of the above were included in this meta-analysis (Fig 1)[2,3,5,20–27]. The characteristics of the included studies are presented in Table 5. Among these studies, 8 were retrospective cohort studies, one was prospective cohort studies, and two were case-control studies. The number of subjects ranged from 105 to 1275, the mean age ranged from 46.5 to 55.0 years old, and the duration of the studies ranged from 4.75 to 10 years. The prevalence of smoking in these studies was 47.8% (95%CI, 46.2% to 49.4%). Assessment of study quality based on Newcastle-Ottawa quality assessment scale (NOS) was shown in Table 5. The relationship between smoking and risk of postoperative complications after liver transplantation was evaluated in 11 studies, comprising 4631 participants (Table 5). Among these studies, six investigated the effect of smoking on cardiovascular diseases (CVD), five on postoperative de novo malignancies, five on hepatic artery thrombosis (HAT), and four on biliary complications. The pooled results showed that smoking had no significant effect on HAT (RR, 1.33; 95% CI, 0.91–1.94; I2 = 0.0%, p = 0.479; Fig 2), and biliary complications (RR, 1.12; 95% CI, 0.90–1.40; I2 = 0.0%, p = 0.561; Fig 3). However, significant association was found between smoking and CVD (RR, 1.31; 95% CI, 1.03–1.67; I2 = 0.0%, p = 0.602; Fig 4), and postoperative de-novo malignancies (RR, 1.90; 95% CI, 1.12–3.22; I2 = 67.4%, p = 0.015; Fig 5). The sensitivity analysis identified that the results remained stable by excluding any single study from the analysis. No publication bias was found in the included studies (p> 0.05).

**Table 5:** Characteristics of studies included in this meta-analysis.

author	year	country	Study design	Sample size (smoker/non-smoker)	Mean age(year)	Gender(male %)	Follow up	Outcome	Quality score
Fussner et al	2015	USA	Cohort	455 (232/223)	51.8	64	NP	CVD	7
Herrero et al	2005	Spain	Case-control	187 (60/127)	55	73	5.4 years	Malignancy	5
Riyaz et al	2013	Pakistan	Cohort	174 (80/94)	52.2	NP	NP	HAT; CVD	6
Perney et al	2013	France	Cohort	105 (79/26)	51.9	80	NP	HAT; CVD; Biliary complication	7
Carenco et al	2015	France	Cohort	465 (280/185)	50.4	74.4	7.8 years	Malignancy	6
Watt et al	2009	USA	Cohort	798 (NP)	49.4	55.5	10 years	Malignancy	7
Coss et al	2011	USA	Cohort	230 (136/94)	50.7	50.7	8.2 years	CVD	8
Heide et al	2009	Netherlands	Cohort	401 (59/236)	46.5	49.1	8.6 years	HAT; CVD; Malignancy	7
Mathur et al	2010	USA	Cohort	409 (249/160)	50.9	63.8	NP	Biliary complications	7
Mangus et al	2015	USA	Cohort	1275 (602/673)	54	74.5	4.7 years	CVD; Biliary complications	6
Leithead et al	2008	UK	Case-control	132 (55/77)	51.4	48.4	8.8 years	HAT; Malignancy	8

Abbreviations: CVD, cardiovascular disease; HAT, hepatic artery thrombosis; NP, not reported.

## Discussion

Advances in surgical techniques and improvement in immunosuppressive therapies have extended graft longevity in liver transplant recipients. Liver transplantation is now considered to be the most effective treatment for patients with end-stage liver disease[28]. The demand for liver transplantation has markedly increased during the past decade. Due to the scarcity of organs available for transplantation, there has been a major increase in the number of patients on transplant waiting lists. The number of patients dying while on the waiting list also increased rapidly[29–31]. Therefore, it is critical to maximize the chances of positive outcomes for liver transplant recipients. Abstinence from smoking is a controversial criterion for liver transplant candidate selection and has been inconsistently applied. Despite the well-known adverse effects of cigarette smoking, its role in early complications following liver transplantation remains inconclusive. In the current analysis of the clinical data of 162 liver transplant recipients in our center, we did not find a significant association between cigarette smoking and immediate major complication following liver transplantation. The incidence of post-transplant biliary complications, hepatic artery thrombosis and in-hospital mortality was comparable among ex-smokers, active smokers and non-smokers. There was no statistical difference in length of hospital stay and ICU stay among the three groups either. Furthermore, the results remain consistency when we compared early postoperative complications according to pack years. The meta-analysis of 11 published studies also showed that smoking had no significant impact on hepatic artery thrombosis (RR, 1.33; 95% CI, 0.91–1.94) and biliary complications (RR, 1.12; 95% CI, 0.90–1.40).

It needs to point out that there are a lot of discrepancies in the reported effects of cigarette smoking on immediate postoperative complications following liver transplantation. The null results found in our cohort study are not in complete agreement with some previous studies. For instance, an increased incidence of vascular complications following liver transplantation has been reported in smokers previously. Pungpapong et al found that the incidence of vascular complications increased from 8% in non-smokers to 17.8% in smokers after liver transplantation[32]. Van der Heide et al. also showed that hepatic artery thrombosis following liver transplantation occurred more frequently in smokers (15%) than nonsmokers (7%)[2]. However, considering the small number of smokers in whom vascular complications developed in these studies (i.e., 29 of 163 smokers in Pungpapong’s study and 9 in 59 smokers in Van del Heide’s study), these results could not be generalized. As a matter of fact, the pooled results of our meta-analysis, which included 11 published studies and a total of 4631 patients, shows no association between cigarette smoking and post-transplant vascular complications. Given that the upper bounds of the 95% CI for the RR of vascular complications associated with cigarette smoking in the meta-analysis, any significant increase in the risk of vascular complications due to smoking can be ruled out. These results are in line with the results of several previous studies. Perney et al found that smokers did not exhibit more post-LT complications than never-smokers[3]. The incidence of hepatic artery thrombosis after liver transplantation was 16.5% in smokers and 19.2% in non-smokers. Similar results were also reported by Leithead et al, which found the incidence of hepatic artery thrombosis after liver transplantation has no statistical difference between smokers (10.9%) and non-smokers (9.1%)[27].

Smoking is a leading cause of premature mortality worldwide. Tobacco use at the time of liver transplant assessment has been shown to be associated with increased all-cause mortality post-transplant[12,26]. However, the increased mortality rate post liver transplant in smokers appears to be non-graft-related. Therefore, the decreased survival post-transplant in smokers was attributed to the negative health implications of cigarette smoking in general, not specifically in the liver transplant recipients. Our current study shows that active smokers fare relatively well immediately after liver transplantation. It is therefore unethically to exclude active smokers from undergoing liver transplantation. Nevertheless, the meta-analysis did show that active smokers had an increased risk for cardiovascular diseases and de-novo malignancies after liver transplantation. In this regard, smokers should still be encouraged to quit before and after liver transplantation.

A few limitations should be noted in interpreting the results from this study. Because of the difficulty in collecting accurate information on occasional smoking and second-hand smoking, we did not include them in the analysis. The focus of this study was to investigate the impact of pre-transplant smoking on early complications after liver transplantation. Our findings cannot be used to explain the effect of post-transplant smoking on long-term outcomes of liver transplantation. As the retrospective nature of the study, the results of this retrospective cohort study are subject to biases related to the adjustment for confounders. Even though adequate controls were used, potential bias remained in the analyses because of unmeasured or unknown confounders. In addition, only a single center’s transplant population data was used in this study, therefore the sample size was small and the incidence of post-transplant mortality and morbidity was low. Regarding the meta-analysis, most of the studies included were retrospective studies; the RRs obtained in this study might be inherently biased by various factors. Although we did not find apparent bias in our meta-analysis, it is difficult to completely rule out the potential publication bias due to the limited number of studies included. Therefore, adequately designed prospective studies in larger cohorts of patients are needed to get a more precise estimate on the prognostic role of cigarette smoking in patients receiving liver transplantation.

In conclusion, we studied the effects of cigarette smoking on early mortality and morbidity after liver transplantation in our center and conducted a meta-analysis of results from other relevant published studies. We found that there is not enough evidence supporting an association between cigarette smoking and early mortality and morbidity after liver transplantation. However, considering the long-term health benefits of smoking cessation, smokers should still be encouraged to quit before and after liver transplantation.

## Supporting information

S1 Table The PRISMA checklist. (DOC)
